# Locked plating of geriatric olecranon fractures leads to low fixation failure and acceptable complication rates

**DOI:** 10.1016/j.jseint.2021.02.013

**Published:** 2021-04-16

**Authors:** Kelsey L. Wise, Sarah Peck, Lauren Smith, Chad Myeroff

**Affiliations:** a University of Minnesota Department of Orthopaedic Surgery, Minneapolis, MN, USA; b Regions Hospital Department of Orthopedic Surgery, Saint Paul, MN, USA; c TRIA Orthopedic Center, Woodbury, MN, USA

**Keywords:** Geriatric, Olecranon, Early mobilization, Locking plate, Implant, Failure mitigation

## Abstract

**Hypothesis:**

The purpose of this study was to report the rate of major complications in patients with geriatric olecranon fractures managed operatively with a locking plate. Secondary objectives included minor complications, as well as pain and range of motion at the final follow-up. We hypothesized that these patients have a low rate of complications as well as low pain and satisfactory elbow range of motion at the final follow-up.

**Materials and Methods:**

A retrospective review of isolated geriatric olecranon fractures presenting from 2006 to 2019 was performed at a single level I trauma center. Inclusion criteria were ≥75 years of age, operative management with a locking plate, and clinic follow-up at least until evidence of radiographic union or a major complication. Exclusion criteria included nonoperative management, insufficient follow-up, and absence of locking plate in surgical technique. Variables examined included demographic information, Charleston comorbidity index, American Society of Anesthesiologists score, living independence, gait assistance, mechanism of injury, open vs. closed fracture, Mayo radiographic classification, Arbeitsgemeinschaft für Osteosynthesefragen classification, time to surgery, implant type, presence of triceps offloading suture, length of postoperative immobilization, date of radiographic union, range of motion at the final follow-up, pain visual analog scale score at the final follow-up, major and minor complications, and return to the operative room. A major complication was defined as a return to the operative room for deep infection or loss of fixation (displacement of fracture >5 mm). A minor complication was defined as any other complication.

**Results:**

A total of 65 patients ≥75 years of age with olecranon fractures were identified. Of these, 36 patients met inclusion criteria with an average follow-up of 23 weeks (range 5-207). The mean length of immobilization was 13 days (range 0-29 days). Thirty-two of 36 (88.8%) patients achieved radiographic evidence of union at an average of 8.9 weeks (range 5.3-24.1 weeks). There were 4 remaining patients who underwent secondary intervention before primary union representing an 11.1% major complication rate including 2 deep infections (5.6%) and 3 failures of fixation (8.3%). There were 7 minor complications in 5 of 36 (13.9%) patients. At the final follow-up, the average visual analog scale score was 2.6 (range 0-6), the average elbow arc of motion was 120° (range 70-147°), and mean pronation/supination was 85°/84° (range 45-90°/45-90°).

**Conclusion:**

Geriatric olecranon fractures are a challenging orthopedic problem with remaining controversy regarding ideal treatment. Despite advancement in geriatric fracture care, there is scant literature on the outcomes of locked plating technology in geriatric olecranon fractures. This study supports use of operative anatomic fixation with precontoured locked plates and early mobilization with an acceptable failure rate.

Olecranon fractures comprise almost 20% of proximal forearm fractures and 8%-10% of all elbow fractures.^16^^,^^39^ These fractures commonly occur from ground-level falls, particularly in elderly individuals. Surgical fixation with tension band wiring (TBW) or plate fixation is the gold standard for displaced olecranon fractures.^3^^,^^27^^,^^34^^,^^37^^,^^43^ Anatomic reduction with a stable fixation construct allows early mobilization and rehabilitation compared with conservative treatment and alternative implants in young, active patients.^1^

Elderly patients who sustain olecranon fractures tend to be women, independent, and medically well. Despite an increasing incidence, controversy continues between conservative and surgical management of olecranon fractures in the geriatric population.^12^^,^^16^ Osteoporotic bone, upper extremity weight-bearing dependence, poor soft-tissue envelope, and medical frailty predispose this population to higher risk of complications, namely fixation failure.^15^^,^^19^^,^^23^^,^^24^^,^^29^^,^^42^ Despite advances in technique and outcomes in many other injuries, these factors have driven focus toward conservative treatment in the elderly patients faced with an olecranon fracture. Even contemporary literature is dominated with nonoperative outcomes, particularly in Europe where acceptable short- and long-term outcomes have been reported in elderly patients after nonoperative management of isolated olecranon fractures.^15^^,^^20^^,^^33^^,^^42^

Nonsurgical treatment has a role in the ultralow demand and medically unwell patients. Acceptable short- and long-term outcomes have been reported in such cases but with a reported rate of subjective and objective elbow extension weakness of 17%-35% and a nonunion rate of 78%-79%.^15^^,^^20^^,^^33^^,^^42^ However, healthier and more active geriatric patients may have compromised outcomes with nonoperative management, as the loss of active elbow extension strength may compromise the ability to rise from a seated position and mobilize with an assistive device.^4^ TBW dominated previous generations of fracture care but has proven to be clinically insufficient, with double the complication rates compared with conventional plates.^14^^,^^41^ Despite mechanical inferiority, all geriatric olecranon fracture trials to date use TBW as the surgical comparison.^14^^,^^41^ It has yet to be demonstrated that good outcomes can be achieved with operative fixation of displaced olecranon fractures in active elderly patients using modern fracture care principles. Precontoured locking plates offer superior fixation strength compared with other methods as a result of the fixed angle construct.^38^ The primary aim of this study was to report the rate of major complications, including fixation failure or deep infection requiring return to the operating room, in patients with geriatric olecranon fractures managed operatively with a locking plate and early mobilization. The secondary aims were to (1) report the rate of minor complications (which include any complication other than a major complication), (2) report the VAS pain level at the final follow-up, and (3) report the elbow range of motion (ROM) at the final follow-up. We hypothesize that in geriatric patients undergoing surgical fixation of an olecranon fracture with a precontoured locking plate, there is a low rate of major and minor complications. In addition, we hypothesize that patients will have low VAS pain levels and satisfactory elbow ROM at the final follow-up.

## Materials and methods

A retrospective review of geriatric (age >75 years) olecranon fractures presenting from 2006 to 2019 was performed at a single level I academic urban trauma center. The inclusion and exclusion criteria are shown in Table I. The primary outcome measure was the presence of a major complication. A major complication was defined as a return to the operative room for deep infection or loss of fixation (displacement of fracture >5 mm). A deep infection met the criteria described by Horan et al.^25^ Secondary outcome measures included minor complications (any complication other than a defined major complication), and both ROM and pain visual analog scale scores obtained at the final follow-up. Patient charts were reviewed from date of injury to present time to maximize inclusion of all complications.

**Table I:** Inclusion and exclusion criteria for the trial.

Inclusion criteria	Exclusion criteria
≥75 yr of age	Nonoperative management
Operative management with a locking plate	Insufficient follow-up
Clinic follow-up at least until evidence of radiographic union or a major complication (return to the operating room for loss of fixation or deep infection)	Absence of locking plate in surgical technique

Demographic data included age, gender, smoking, body mass index, living independence, gait assistance, hand dominance, and mechanism of injury. The American Society of Anesthesiologists grade^13^ and the Charlson comorbidity index (CCI)^8^ were obtained for all patients.

All fractures were classified using the Mayo classification^30^ and the Arbeitsgemeinschaft für Osteosynthesefragen classification^1^ by the 2 most senior authors (CM and LC) using standard anteroposterior and lateral radiographs of the elbow. When classification was not unanimous, final type was determined through utilization of collaborative review and unanimous consensus. Other data points collected included open vs. closed fracture, time to surgery, implant type, presence of triceps offloading suture, length of postoperative immobilization, and date of radiographic union.

**Figure 1:**
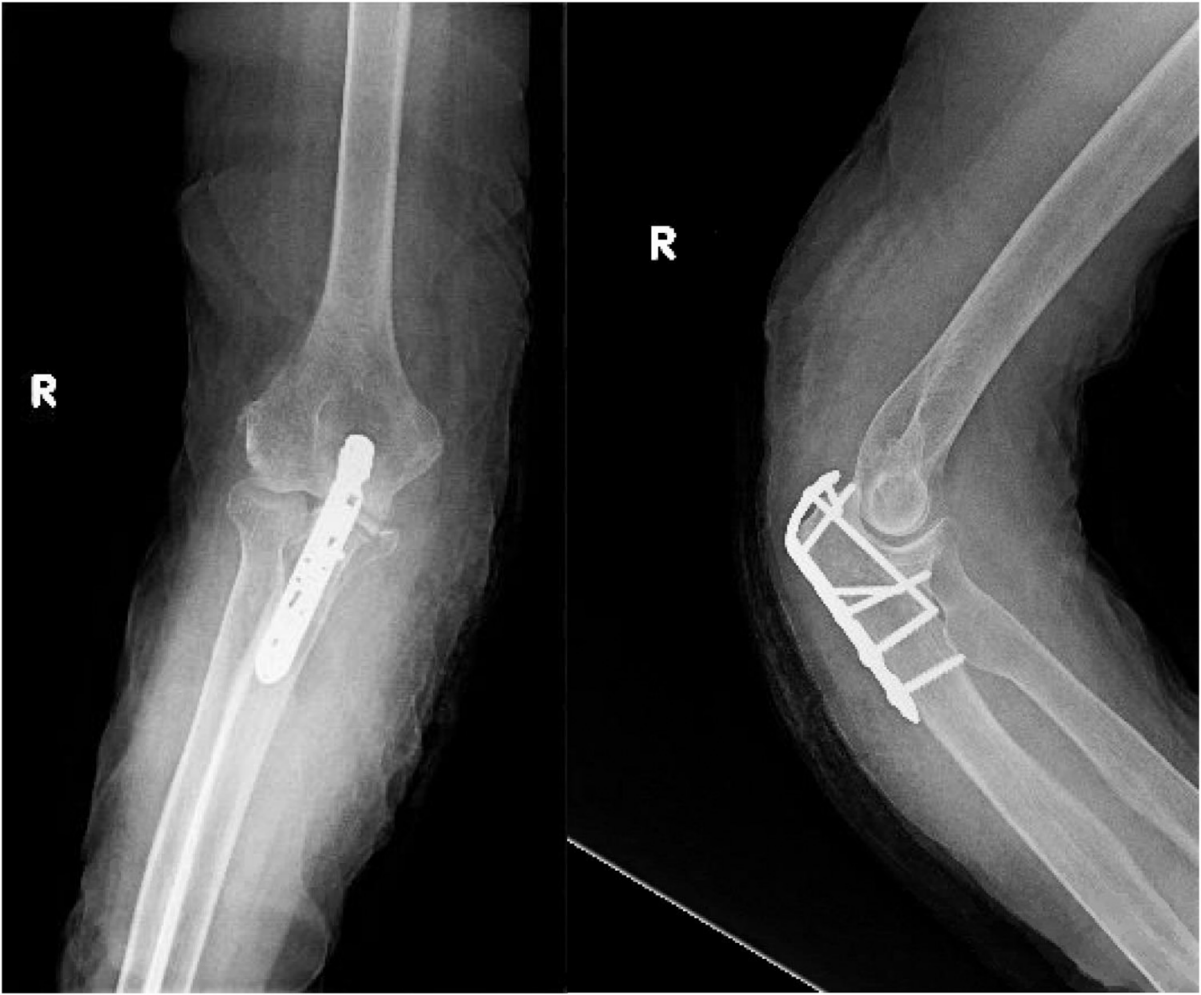
Locked plate fixation for olecranon fracture in a geriatric patient. Postoperative AP and lateral radiographs of an 86-year-old female patient. *AP*, anteroposterior.

Anteroposterior and lateral radiographs of the elbow (Fig. 1) were examined by the senior author (CM) to assess the quality of the reduction, failure of fixation, loss of reduction involving redisplacement of the articular surface by > 2 mm, and progression to union. The reduction was deemed acceptable if the articular surface was reduced to within 2 mm.^27^ Union was defined as endosteal healing with ≥ 75% organized trabecular bridging at the fracture site.^40^ The senior author performed radiographic review of all patients to determine presence and timing of union. To be included, patients had to be followed up clinically until at least radiographic union or major complication.

## Results

A total of 65 patients ≥75 years of age with olecranon fractures were identified. After exclusions, 36 patients were included with a mean follow-up of 23 weeks (5-207; standard deviation [SD] 34).

**Figure 2:**
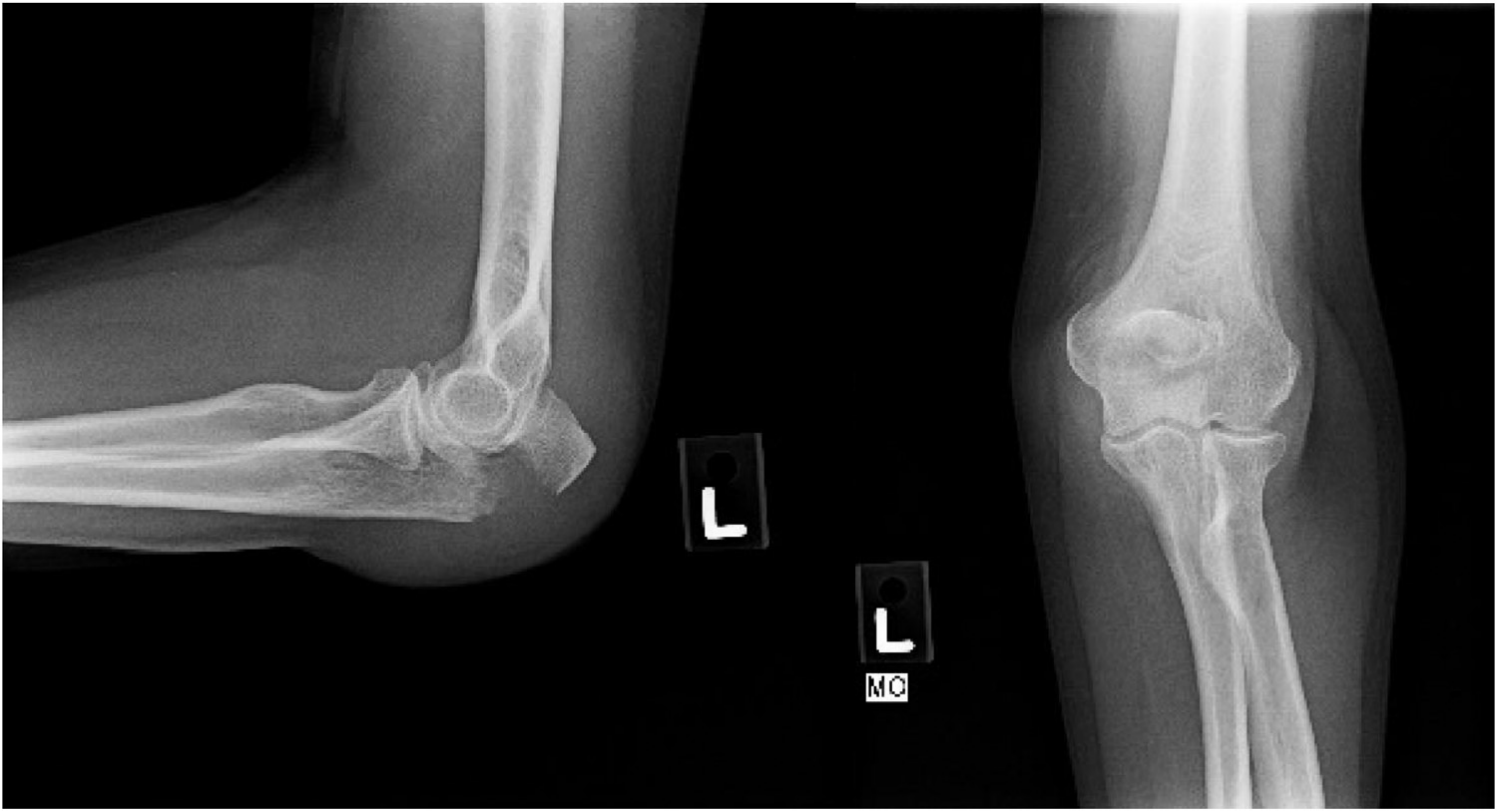
Mayo 2a, AO 2u1b1d. 76-year-old female presenting with a Mayo 2A (displaced, noncomminuted) and AO classification 2u1b1s (partial articular olecranon fracture, simple) olecranon fracture after a ground-level fall. *AO*, Arbeitsgemeinschaft für Osteosynthesefragen.

**Figure 3:**
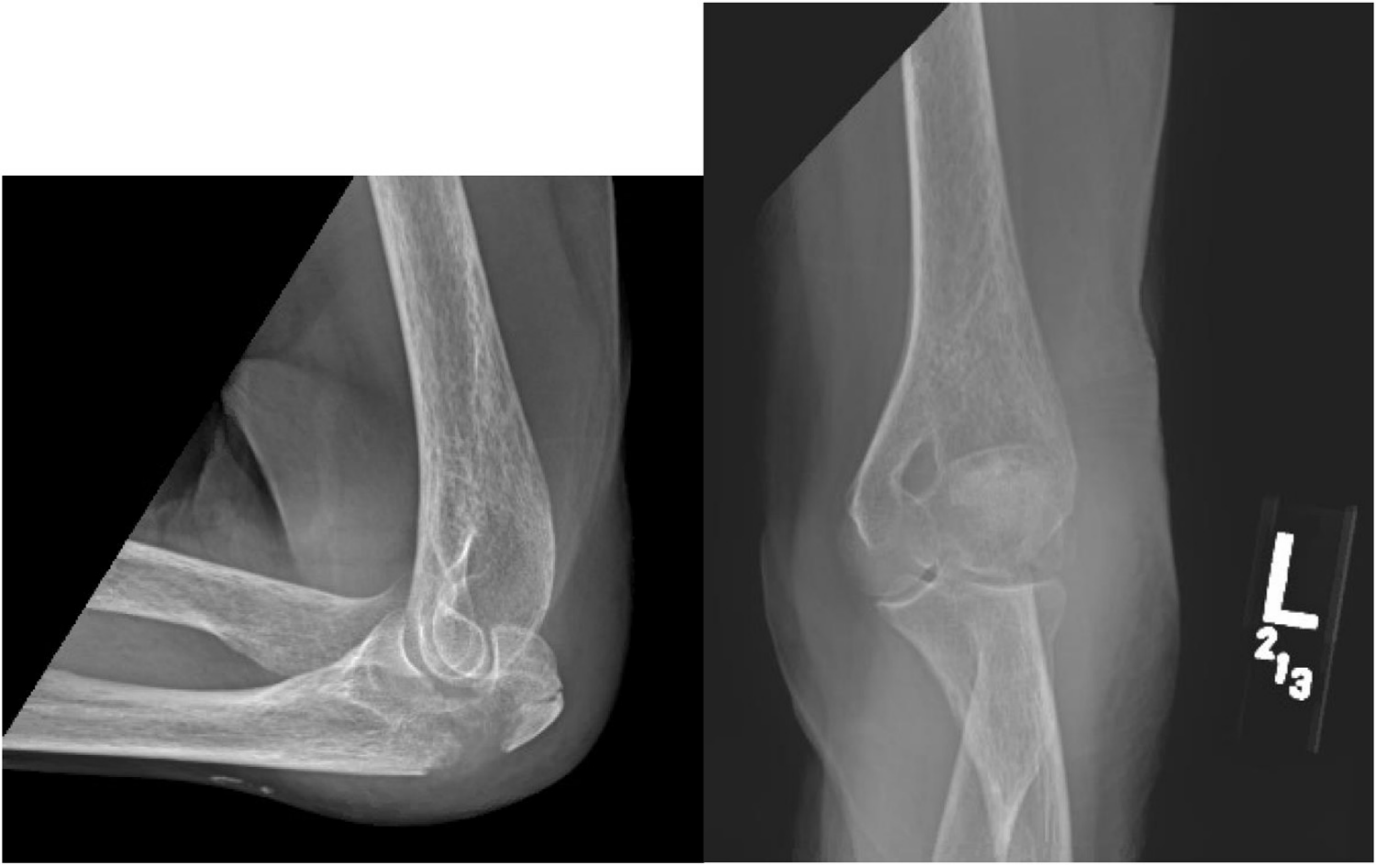
Mayo 2b, AO 2u1b1e. 85-year-old female presenting with a Mayo 2A (displaced, comminuted) and AO classification 2u1b1s (partial articular olecranon fracture, multi-fragmentary) olecranon fracture after a ground-level fall. *AO*, Arbeitsgemeinschaft für Osteosynthesefragen.

The overall mean age of patients was 83.8 years (75-96; SD 5.4), and 26 of 36 (72.2%) were women. Mean body mass index was 22.6 (16.73-30.0; SD 3.5). The mean CCI with no age adjustment was 1.8 (0-5; SD 1.5). The mean age-adjusted CCI was 5.6 (3-8; SD 1.6). Most patients were of American Society of Anesthesiologists grade III (22 of 36, 61%) or II (13 of 36, 36%). Most patients were nondiabetic (31 of 36, 81.6%) and former smokers or nonsmokers (34 of 36, 94.4%). The majority of patients lived independently (28 of 36, 77.8%) and walked with no assistive devices (26 of 36, 72.2%). The most common mechanism of injury was a ground level fall (32 of 36, 88.9%). Half of the patients injured their nondominant upper extremity (16 of 36, 50%). Fifteen patients had concomitant injuries at presentation (41.7%) (Table II). All patients had Mayo 2A (19 of 36, 52.7%) (Fig. 2) or 2B (17 of 36, 47.2%) (Fig. 3) fracture patterns. The most common Arbeitsgemeinschaft für Osteosynthesefragen fracture patterns were 2U1B1D (17 of 36, 47.2%) (Fig. 2) and 2U1B1E (16 of 36, 44.4%) (Fig. 3). The mean time from injury to surgery was 4.8 days (range 1-21 days). Six of the cases (16.7%) used triceps offloading suture. The mean length of immobilization was 13 days (range 0-29 days). Thirty-two patients achieved radiographic evidence of union (32 of 36, 88.8%) at an average was of 8.9 weeks (range 5.3-24.1 weeks). The remaining 4 of 36 patients did not go on to “primary union,” as they required early reintervention before establishing bony union. Of those who did not have an early major complication, union rate was 100% (32 of 36).

**Table II:** Concomitant injuries and management.

Patient	Injury	Management
1	Rib fractures	Nonoperative
2	Closed head injury	Nonoperative
3	Nondisplaced proximal phalanx fracture of foot	Nonoperative
4	Pleural effusion	Nonoperative
5	Femoral neck fracture	Cephalomedullary nail
6	Intertrochanteric femur fracture	Cephalomedullary nail
7	Femoral neck fracture	Dynamic hip screw
8	Subdural hematoma	Nonoperative
9	Intertrochanteric fracture	Dynamic hip screw
10	Super and inferior rami fractures, sacral ala fracture	Nonoperative
11	Sacral U-type fracture	Percutaneous screw fixation
12	Femoral neck fracture	Hemiarthroplasty
13	Peri-implant distal third femoral shaft fracture	Open reduction and internal fixation
14	Femoral neck fracture	Cephalomedullary nail
15	Femoral neck fracture	Hemiarthroplasty

### Primary outcome

There were 5 major complications in 4 patients (4 of 36; 11.1%), all of which required return to the operative room before bony union, including 2 deep infections (2 of 36, 5.6%) and 3 failures of fixation (3 of 36, 8.3%) (Table III). Table IV compares demographic data of the 4 patients with major complications to the entire series of patients. While statistical comparisons cannot be drawn, the failure group had a higher average age (86.8 vs. 83.8 years), assisted living arrangement (75% vs. 22.2%), gait assistance with walker (75% vs. 27.8%), presence of other injuries (polytrauma) (75% vs. 41.7%), body mass index (25 vs. 22.6), CCI (2.3 vs. 1.8), American Society of Anesthesiologists score (3 vs. 2.6) than all comers.

**Table III:** Major complications and management.

Major complication	Patient	Time from surgery (d)	Management
Deep infection	1	37	Antibiotics, irrigation, and debridement
	2	14	Antibiotics, revision open reduction, and internal fixation
Failure of fixation	2	14	See aforementioned information
	3	41	Revision open reduction and internal fixation
	4	13	Revision open reduction and internal fixation

**Table IV:** Demographic data of the 4 patients with major failures compared with the entire study sample.

Demographic datapoint	Major failures	All patients
Mean age (yr)	86.8	83.8
Assistive living (%)	75	22.2
Gait assistance with walker (%)	75	27.8
Presence of other injuries (%)	75	41.7
Mean BMI (kg/m^2^)	25.0	22.6
Mean CCI (no age adjustment) (n)	2.3	1.8
Mean CCI (with age adjustment) (n)	6.3	5.6
Mean ASA (n)	3	2.6

*ASA*, American Society of Anesthesiologists; *BMI*, body mass index; *CCI*, Charlson comorbidity index.

### Secondary outcomes

There were 7 minor complications in 5 patients (5 of 36; 13.9%), including elective symptomatic implant removal (2 of 36, 5.6%), malreduction (<5 mm) (2 of 36, 5.6%), loss of fixation (<5 mm) that did not result in return to the operating room (2 of 36, 5.6%), and heterotopic ossification (1 of 36, 2.8%) (Table V). At the final follow-up, the mean visual analog scale score was 2.6 (0-6; SD 2.1). The mean elbow arc of flexion was 120° (70-147°; SD 20.0°). The mean forearm pronation was 85° (45-90°; SD 10.1°), and the mean forearm supination was 84° (45-90°; SD 10.2°).

**Table V:** Minor complications.

Complication	Number (n)
Implant removal	2
Malreduction	2
Loss of fixation with no return to the OR	2
Heterotopic ossification	1

*OR*, operative room.

## Discussion

Management of olecranon fractures in elderly is challenging because of proposed surgical and anesthetic risks as a result of frailty, comorbidities, poor soft-tissue envelope, and osteoporotic bone.^42^ This study found low complication rates with the use of precontoured locked plates in a geriatric population. In addition, patients had low pain levels and satisfactory elbow ROM at the final follow-up.

This study has several important limitations. This was a retrospective series with a small cohort of patients, many of whom had follow-up of less than 1 year. However, we surmise that the majority of these patients did not follow up longer because they were asymptomatic, having already achieved evidence of radiographic union, or had deceased, which is a reality of follow-up of elderly patients with inherent medical comorbidities. No statistical models were able to be used to identify patterns in patients with complications, major or minor, as the study was underpowered. Another area for improvement would be the inclusion of outcome scores such as the disability of the arm, shoulder, and hand (DASH),^21^^,^^26^ the Mayo Elbow Score,^31^ and the Broberg and Morrey score^6^ to provide additional data and allow comparison with other studies. Given the high complication and failure rate with TBW for geriatric olecranon fractures and the increasing physical demands of our aging population, we feel a randomized controlled trial comparing locked plating vs. conservative treatment is warranted. This could help identify the gold standard treatment and further clarify the ideal subgroups for each treatment.

Acceptable outcomes have been reported with nonoperative management of geriatric fractures.^10^^,^^14^^,^^15^^,^^42^ Veras Del Monte et al^42^ retrospectively reviewed 12 patients of mean age 81.8 years with olecranon fractures managed with elbow immobilization for an average of 4.1 weeks. Mean arc of flexion at the final follow-up was 129° and pronation/supination 83.5°/83.5°. Eight patients had good outcomes, whereas 4 had fair or poor. Duckworth et al^15^ retrospectively evaluated 43 patients of mean age 76 years managed conservatively with collar and cuff or an above-the-elbow plaster cast at 60-90° of flexion for a mean of 4 weeks. At an average of 4 months, mean arc of elbow flexion was 109° and mean forearm pronation/supination was 79°/80°. The average DASH score was 2.9; 31 patients had excellent or good outcomes, whereas 12 patients had fair or poor outcomes. They reported a 78% nonunion rate, and 17% of patients had noticeable push off weakness.^15^ Gallucci et al^20^ retrospectively reviewed 28 patients >70 years of age treated conservatively and similarly found functional ROM (15-140°), DASH 15, Mayo Elbow Performance Index 90, 79% nonunion, and 35% of patients had objective triceps weakness. Our study found comparable ROM to the patients from these 2 studies. An important distinction of our study is the shorter period of immobility, which could potentially lead to quicker return to activities of daily living, lower pain levels, and long-term ROM.

Anatomic reduction of displaced olecranon fractures allows early mobility, prevents stiffness, and minimizes the risk of secondary arthrosis.^34^ TBW and plate fixation are the most popular surgical options. Alternatives, which have shown acceptable outcomes in small studies in young bone, include fracture fragment excision with triceps tendon advancement,^5^^,^^22^ suture anchor fixation,^4^^,^^7^ and intramedullary nailing.^2^ TBW is thought to convert tensile forces across the fracture as a result of the pull of the triceps into compressive forces at the joint surface and can be used for simple, transverse fractures.^34^ A major setback of this procedure is the high incidence of reduction loss in elderly patients with porotic bone.^10^^,^^14^^,^^19^^,^^24^^,^^29^^,^^36^ Plating is another option for both stable and unstable displaced fracture patterns. Precontoured locking plates are advantageous compared with traditional plates in unstable fractures, and there is decreased chance of narrowing the radius of the sigmoid notch from overreduction, which can lead to malunion or joint incongruity and secondary stiffness and arthrosis.^34^ Such plates allow more compression and polyaxial screw fixation, which is biomechanically superior to colinear screws seen in traditional plates.^18^ Locking technology has proven its worth in osteoporotic fractures in many other metaphyseal fractures.^9^^,^^11^^,^^28^^,^^32^^,^^35^ Prospective randomized studies comparing TBW and plate fixation found a higher number of complications, including loss of fracture reduction and prominent implant, in the TBW group.^15^^,^^27^ Although the randomized study published by Duckworth et al^16^ in 2017 had a significantly younger patient population in the TBW group (43 vs. 52 years of age; *P* = .028), they showed double the rate of loss of reduction (27% vs. 13%; *P* = .206) and removal of implant rate (59% vs. 22%; *P* = .021) for TBW. The patients in these studies were considerably younger than the patients in our study (mean ages of 30.9 and 43 vs. 83.8). It is likely that geriatric patient populations, such as the ones included in our cohort, would see even higher rates of complications with TBW as a result of osteoporotic bone.

Despite contemporary disfavor for TBW in osteoporotic populations, its use is ubiquitous, even in the most modern literature on the management of elderly patients with olecranon fractures.^10^^,^^14^^,^^17^ Umer et al^41^reported in 79 olecranon fractures in elderly patients (>70 years of age) treated with TBW (57 of 79; 56% male patients). Results were poor with 14% wounds, 16% persistent pain, 44% metal problems, 19% removal implant, and 75% loss of motion.^41^ In 2017, Duckworth et al^14^ published a prospective randomized trial comparing nonoperative and operative treatment of olecranon fractures in elderly patients aged 75 years and older. Owing to high failure rate in the operative group, the study was stopped owing to loss of equipoise by the treating surgeons. Of the patients managed surgically, there was an 81.8% complication rate (13 complications in 10 patients) in the operative group vs. 14.3% in the nonoperative group (*P* = .013). The surgical complications included 6 of 11 (54.5%) loss of reduction, 3 of 11 (27.3%) removal of implant, and 1 of 11 (9.1%) excision of a sinus draining wound. Notably, the surgeons used TBW in 9 of 11 (81.8%) of cases and nonlocking plates in the remaining 2 of 11 (18.2%), and patients were immobilized for 10-14 days. No locking plates were used in this geriatric cohort.

Comparing our cohort with those randomized by Duckworth et al,^14^ patients had similar fracture characteristics (Mayo 2a and 2b) and were of similar age (≥75 years; mean 83 years), although their operative cohort was older than the nonoperative cohort (85 vs. 80 years), which would bias the risk of failure toward the operative cohort. In the study by Duckworth et al,^14^ no locking plates were used, and the minority (4 of 11; 36.3%) were performed by the attending. They reported an 81.8% complication rate after open reduction internal fixation (including 6 of 11 [55%] mechanical failure) vs. 14.3% nonoperative (*P* = .013). Nonunion occurred in 7 of 11 (63.6%) nonoperative patients and 2 of 11 (18.2%) operative patients, whereas we did not experience any nonunions in our cohort that did not require early reintervention (32 of 32; 100%). DASH (23 vs. 22; *P* = .763) and Bromberg and Morrey (*P* > .05) showed similar functional outcomes between operative and nonoperative treatments at all time points. While functional scores were similar between operative and nonoperative arms, more flexion was achieved (129° vs. 106°; *P* = .049) in those undergoing surgery, which compares with our findings. Their study did not show inferiority of surgery but did call into question the risk of complications, namely failure of fixation which occurred in 6 of 11 (54.5%) cases. They chose to use predominantly TBW because “adequate screw fixation in a small osteoporotic proximal fragment is difficult to achieve and it is unlikely that this would provide a better outcome than non-operative management in these patients.” We would argue the opposite and that locked plating allows more points of fixation, polyaxial screw spread, more compression, a more rigid construct, and earlier functional rehabilitation. Our study demonstrates that locked plate fixation in select patients 75 years of age or older allows early mobilization with a low failure rate and high union rate. Patients were found to have good range of motion and overall low pain scores at the final follow-up. Because the main downfall of surgery in their study was mechanical failure, it can be hypothesized that a randomized study with a lower fixation failure rate using locked plating may be able to show improved overall functional outcomes with surgical management in select elderly patients.

It is important to note that this cohort was not only comprised isolated olecranon fractures.

Many of our patients were patients with polytrauma and more than one-quarter used assistive ambulatory devises before injury. We felt that including such patients makes this a very pragmatic study encompassing the breadth of patients the typical trauma surgeon would consider for operative treatment. Although not powered for statistical analysis, we found that patients in this study who sustained a major complication were qualitatively more likely to require assisted living, use a walker for gait assistance, and present to the hospital with other injuries. This suggests that while high upper extremity demands are often an indication for surgery, this subgroup could be predisposed to failure for the same reasons.

## Conclusion

Geriatric olecranon fractures present a clinical challenge with remaining controversy regarding the ideal treatment. Poor bone quality, tissue frailty, variable compliance, and upper extremity dependence have historically yielded high clinical failures with traditional techniques.^2^^,^^4^^,^^7^^,^^15^^,^^22^^,^^23^ Despite advancement in geriatric fracture care, there is scant literature available on the outcomes of locked plating technology in geriatric olecranon fractures using contemporary principles. This study supports use of operative anatomic fixation with precontoured locked plates and early mobilization in the appropriately selected geriatric patient population, with an acceptable failure rate.

## Disclaimers:

*Funding:* No funding was disclosed by the author(s).

*Conflicts of interest:* The authors, their immediate families, and any research foundations with which they are affiliated have not received any financial payments or other benefits from any commercial entity related to the subject of this article.
